# Analysis of axonal trafficking via a novel live-imaging technique reveals distinct hedgehog transport kinetics

**DOI:** 10.1242/bio.024075

**Published:** 2017-03-15

**Authors:** Joseph R. Daniele, Rehan M. Baqri, Sam Kunes

**Affiliations:** Department of Molecular & Cellular Biology, Harvard University, 16 Divinity Avenue, Cambridge, MA 02138, USA

**Keywords:** Axonal Transport, Hedgehog, Live Imaging, Photoreceptor Axons, Neurodegeneration, Drosophila

## Abstract

The *Drosophila melanogaster* (*Dmel*) eye is an ideal model to study development, intracellular signaling, behavior, and neurodegenerative disease. Interestingly, dynamic data are not commonly employed to investigate eye-specific disease models. Using axonal transport of the morphogen Hedgehog (Hh), which is integral to *Dmel* eye-brain development and implicated in stem cell maintenance and neoplastic disease, we demonstrate the ability to comprehensively quantify and characterize its trafficking in various neuron types and a neurodegeneration model in live early third-instar larval *Drosophila*. We find that neuronal Hh, whose kinetics have not been reported previously, favors fast anterograde transport and varies in speed and flux with respect to axonal position. This suggests distinct trafficking pathways along the axon. Lastly, we report abnormal transport of Hh in an accepted model of photoreceptor neurodegeneration. As a technical complement to existing eye-specific disease models, we demonstrate the ability to directly visualize transport in real time in intact and live animals and track secreted cargoes from the axon to their release points. Particle dynamics can now be precisely calculated and we posit that this method could be conveniently applied to characterizing disease pathogenesis and genetic screening in other established models of neurodegeneration.

## Abstract (precis)

**Summary:** A novel method to directly visualize Hedgehog transport in the photoreceptor neurons of living animals offers unprecedented positional and temporal detail of complex phenotypes in real time.

## INTRODUCTION

Since the discovery of the first eye-specific mutant (*white*) in 1910, the *Drosophila melanogaster* (*Dmel*) visual system has been the focus of innumerable genetic screens, ranging from research in development, intracellular signaling, behavior, and in particular, neurodegenerative disease. In addition to the practical implications, *Dmel* disease models are popular because many (>75%) genes associated with human disease are conserved between fly and human and the basic fundamentals of cell biology are similar between these two species. In particular, the developing visual system has been a model of choice since the fly eye is amenable to genetic disruption and is dispensable for the organism's survival (Lenz et al., 2013; Sang and Jackson, 2005).

Although many eye-specific models of neurodegenerative disease have been developed (Alzheimer's disease, Parkinson's disease and trinucleotide expansion disorders such as Huntington's disease), these studies have mainly used static readouts of phenotype (e.g. biochemistry, immunohistochemistry, pathology, rescue of ‘rough-eye’) (Lenz et al., 2013; Sang and Jackson, 2005). Only a select few of these models have focused on dynamic data such as the velocity, flux, and positional distribution of pathogenically relevant proteins (e.g. PINK1, polyglutamine-containing proteins, Lis1) or organelles in these mutated tissues (e.g. mitochondria) (Liu et al., 2000, 1; Stowers et al., 2002; Wang et al., 2011; Wyan-Ching Mimi Lee et al., 2004). This disparity between static and dynamic data is significant in the field of development especially with respect to the morphogen Hedgehog (Hh), which is trafficked down *Dmel* photoreceptor axons and is integral to eye-brain development. Amongst Hh-expressing tissues (*Dmel* embryo and wing disc, planarian CNS regeneration, and vertebrate central nervous system (CNS), limb, organs, and foregut) developing photoreceptor axons offer a unique and accessible means to study Hh intracellular trafficking (Jiang and Hui, 2008; Yazawa et al., 2009).

Hedgehog (Hh) is a highly conserved secreted morphogen capable of patterning many developing tissues. More recently, Hh signaling has been implicated in both *Dmel* and mouse adult stem cell maintenance and human neoplastic disease (Bale, 2002; Ihrie et al., 2011; Jiang and Hui, 2008; Rojas-Ríos et al., 2012; Scales and de Sauvage, 2009; Varjosalo and Taipale, 2008). While a number of papers have investigated Hh extracellular movement and downstream signaling, little is known about the mechanisms regulating its intracellular transport and secretion. To investigate the nature and factors governing Hh transport we developed a novel method to directly visualize and characterize this process in the photoreceptor axons of live and intact *Drosophila* larvae.

Using visualization of Hh in *Dmel* photoreceptors, we demonstrate the ability to comprehensively quantify the movement and directionality of important cargoes during eye and brain development in a practical and accessible model system. To our knowledge, this is the first instance of dynamic Hh data from neurons that has comprehensively analyzed its transport.

Unlike most model systems of axonal transport, which involve cultured neurons, our data are collected from intact organisms in which the normal environment and interactions with other cell types are preserved. While this Hh system could be applied to many cell biological questions (e.g. protein and/or organelle transport throughout disease progression) we focus on the apical/basal transport of Hh in developing photoreceptors in healthy and disease models, as this is particularly difficult to observe and quantify in other established models of Hh in development (Jiang and Hui, 2008; Yazawa et al., 2009). More broadly, this method could be adapted to additional neurodegenerative disease models (e.g. in the *Dmel* eye) and should enable future research to characterize the role of pathogenic proteins and organelles, candidate drug treatment, apical/basal cell polarity, and axon transport in development in any practical, transparent, and genetically tractable organism.

## RESULTS

**Fig. 1.**
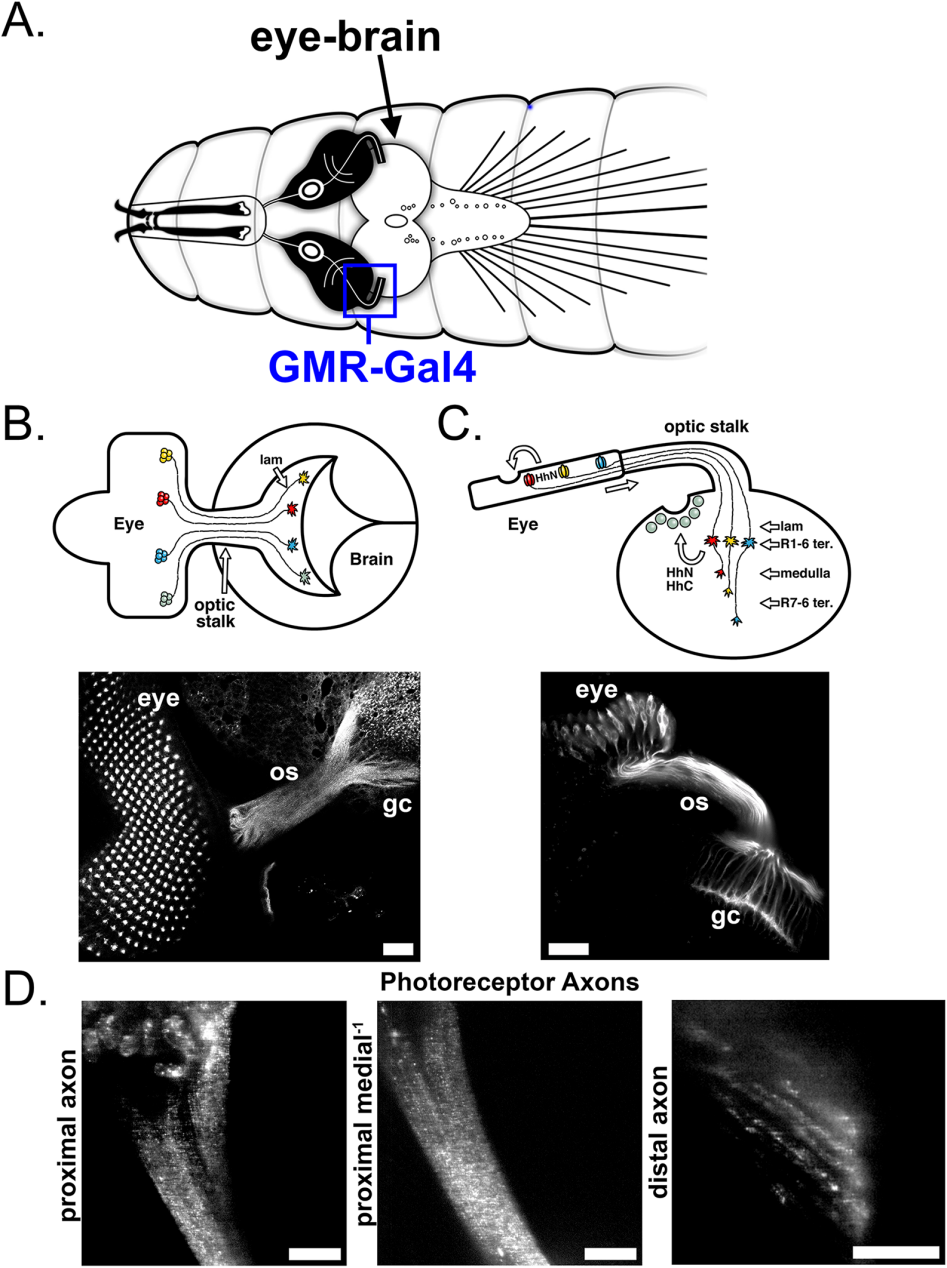
**Anatomy and visualization of Hh in larval *Drosophila melanogaster* neurons.** (A) Schematic of third-instar larval CNS depicting the location of the developing *Dmel* visual system and the *GMR-Gal4* driver which expresses in this tissue. (B,C) The visual system shown from the lateral (B) and horizontal (C) perspective. (B) *Dmel* visual system shown from the lateral perspective. Photoreceptor neurons differentiate temporally with the posterior-to-anterior progression (top, right to left) of the morphogenetic furrow across the eye disc. These neurons project their axons (R1-R8) into the brain through the optic stalk, where they spread to dorsal and ventral retinotopic positions (dorsal is up). Micrograph shows the *Dmel* eye from the lateral perspective. Image is an immunostaining against the eye-specific protein Chaoptin (24B10). Os, optic stalk; gc, growth cone (axon termini). (C) The *Dmel* visual system shown from the horizontal perspective. The R1-R6 axons terminate in the lamina (lam), while R7 and R8 axons terminate in the deeper medulla ganglion. Hh secreted from developing photoreceptor neurons is required for both eye and lamina (brain) development (top). Micrograph shows the *Dmel* eye from the horizontal perspective. As in (B), image is an immunostaining against Chaoptin (24B10). (D) Representative still frames from time-lapse movies of *Dmel* expressing ss::GFP::HhC (HhC) in developing photoreceptors (*GMR-Gal4*>*UAS- ss::GFP::HhC*, see Fig. 1A) at various positions in the axon. HhC punctae can be seen in the proximal axon with the apical photoreceptor cell bodies seen in the top of the micrograph (left). HhC punctae in the proximal and medial axon with the apical side on the top and basal side on the bottom (middle). HhC punctae in the distal axon termini of the photoreceptor (right). Scale bars: 20 µm (B,C), 5 µm (D).

In fly larval photoreceptor neurons the developmental signal Hh is guided to two receptive fields; the apical (retina) and basal (growth cone, GC) ends where secretion of the morphogen is an inductive factor in photoreceptor differentiation and establishment of eye-brain neural connections (Fig. 1A-C) (Huang and Kunes, 1996; Roignant and Treisman, 2009). Hh released apically induces ommatidial development (Fig. 1C,D, left), while Hh transported down the photoreceptor's axon (Fig. 1D, middle) and released in the brain induces the development and specification of postsynaptic target neurons (Fig. 1D, right). This phenomenon has also been described in retinal ganglion cells during rat visual system development (Beug et al., 2011; Dakubo et al., 2003, 2008; Soukkarieh et al., 2007; Wallace and Raff, 1999; Wang et al., 2005). Thus, a balance of apical/basal transport to two receptive fields is integral to the precise timing and specification of the *Drosophila* eye and brain.

Hh undergoes autocleavage from its full-length form (HhU; 46 kDa) to become two products, a cholesterol-modified N-terminal signaling molecule (HhN; 19 kDa) and a C-terminal molecule (HhC; 24 kDa), the full role of which is still unknown (Lee et al., 1994). HhN contains all the information necessary for downstream signaling but when the C-terminal domain (HhC) is mutated or deleted from the full-length protein it is unable to undergo axonal transport. Under these circumstances all HhN is released at the apical (retina) side. The C-terminal domain, however, contains a conserved motif that designates the protein for axonal transport and release at the growth cone. Indeed, HhC (which travels with HhN) is necessary for the proper axonal transport of the N-terminal signaling domain (Chu et al., 2006).

**Fig. 2.**
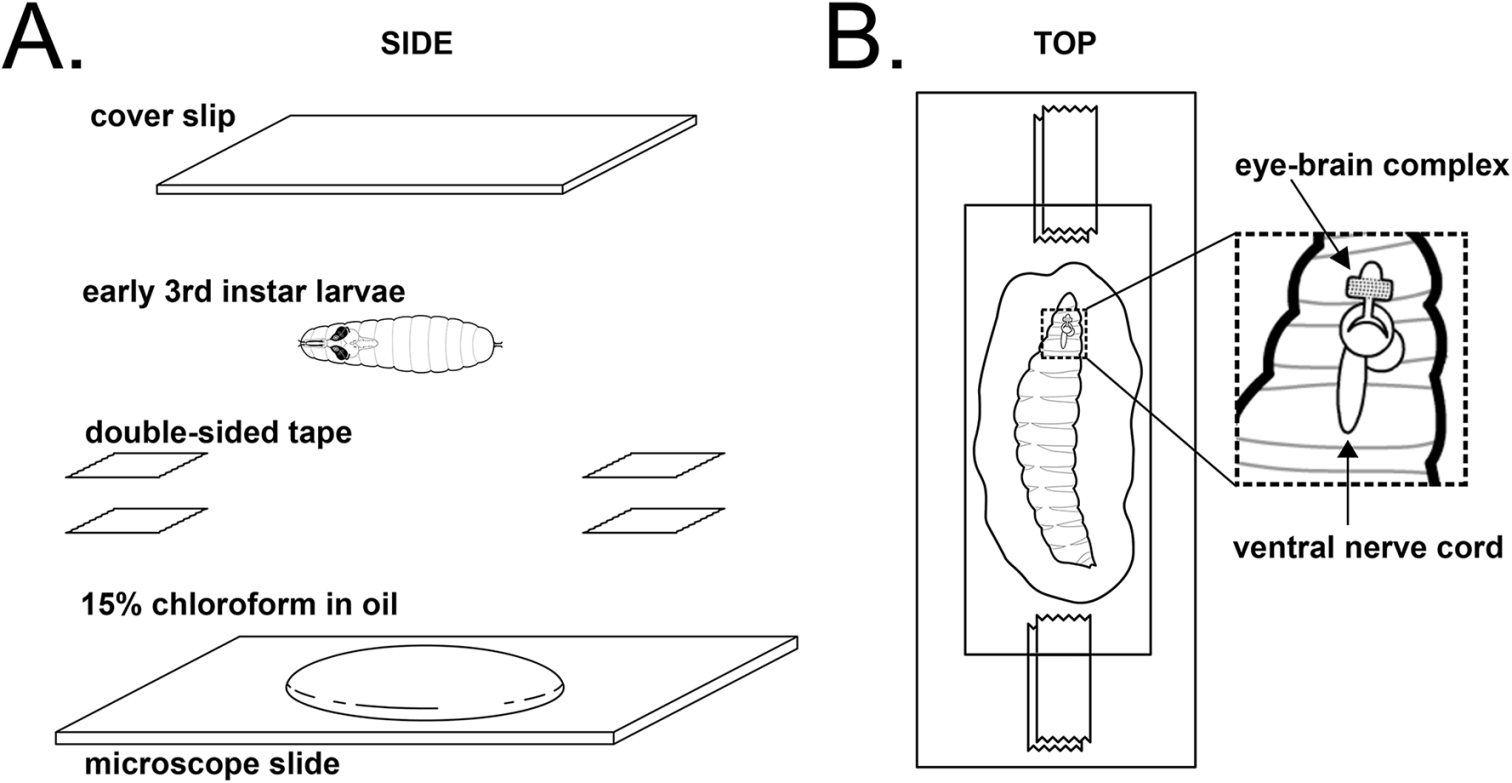
**Experimental setup for live imaging of larval *Dmel* neurons.** (A) Side view schematic for mounting and imaging of live larvae. (B) Top view schematic shown to illustrate proper angle of mounting and intended orientation of larval eye-brain complex. Larvae have been intentionally drawn larger to illustrate anatomy and orientation of eye-brain complex to enable proper positioning.

**Fig. 3.**
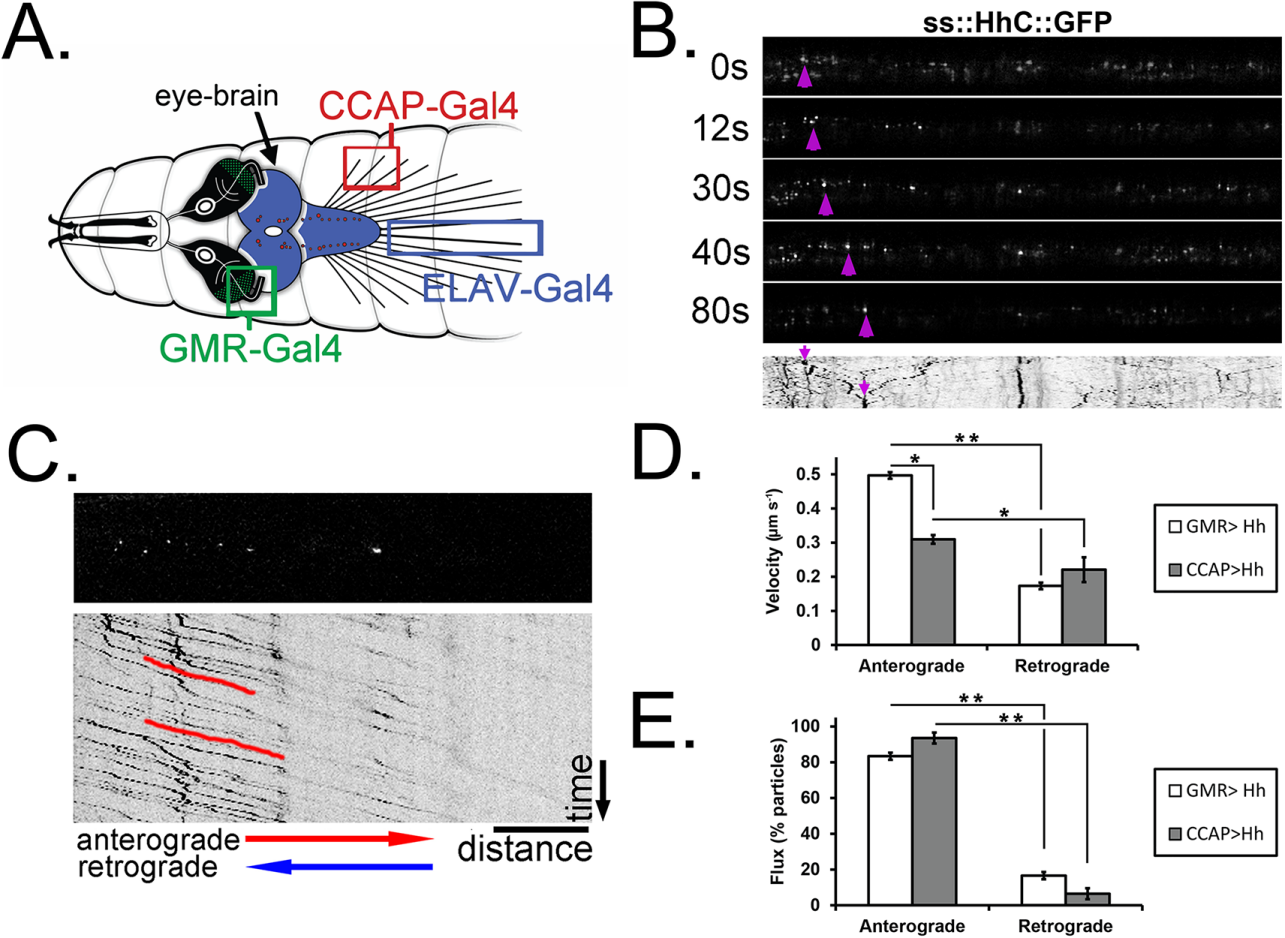
**Quantification of HhC transport along *Dmel* neurons.** (A) Schematic of third-instar larval CNS depicting the distinct axonal populations that were imaged in *ELAV-GAL4* (B) and *CCAP-GAL4* and *GMR-Gal4* animals (C-E). The region of the axon imaged is boxed in the same color as the cell bodies where Hh was expressed. (B) Still frames and accompanying kymograph from a time-lapse series of HhC transport in animal expressing ss::GFP::HhC driven by *ELAV-GAL4* (Luan et al., 2006). The weak *ELAV-Gal4* driver was chosen to illustrate the tracing of a single HhC particle (arrowheads) in an axon. Arrow indicates the kymograph profile of the highlighted puncta (bottom). (C) HhC punctae translocate along the axon in larvae expressing ss::GFP::HhC driven by *CCAP-GAL4* (Movie 1). Accompanying kymograph (bottom) illustrating the transport profile in this axon establishes that HhC is transported almost exclusively in the anterograde direction (red trace). Scale bars: 10 µm (B,C). Movies of ss::GFP::HhC (Movies 2-4) (under *GMR-GAL4* control) in larval photoreceptors are also provided. (D,E) Quantification of anterograde and retrograde velocity (D) and flux (E) of HhC particles in *Dmel* photoreceptors (*GMR-Gal4*, white bars) and motor neurons (*CCAP-Gal4*, grey bars). Values provided are means from ∼8 min of time lapse data using the *CCAP-Gal4* driver [*n*(analyzed traces)=219] and ∼10 min of time-lapse data using the *GMR-Gal4* driver [*n*(analyzed traces)=308]. *P<0.05 and **P<0.01 by two-tailed *t*-test. Error bars are s.e.m.

To determine whether HhC undergoes axonal transport, we observed trafficking via real-time imaging of axons in live *Drosophila* larvae (Fig. 2 and see Materials and Methods for step-by-step directions regarding data acquisition and analysis). Fluorescently tagged HhC (ss::GFP::HhC) was first expressed with *CCAP-GAL4*, which drives expression in a small population of motor neurons that extend axons along segmental nerves (Fig. 3A) (Luan et al., 2006; Vömel and Wegener, 2007). The directionality of transport can be unambiguously determined because sensory axons sharing the nerve are not labeled (Wang et al., 2011). To graphically represent transport behavior we generated kymographs, which denote distance translocated on the *x*-axis versus time on the *y*-axis (Fig. 3B,C) (Miller and Sheetz, 2006). The slope of this trace yields velocity of transport. Such an analysis revealed that velocity of Hh transport was 0.31 µm s^−1^ in the anterograde direction, and 0.22 µm s^−1^ in the retrograde direction (Fig. 3D, grey bars and Movie 1). A similar analysis on HhC expressed in photoreceptor neurons (*GMR-Gal4*; Fig. 3A,D, white bars and Movie 2; Tare et al., 2011) showed a larger disparity between anterograde and retrograde transport, with the velocities calculated at 0.50 and 0.17 µm s^−1^, respectively. The ranges of velocities strongly suggest that Hh is trafficked within motor neuron and photoreceptor axons via microtubule-based fast axonal transport and that kinesin and dynein are its primary molecular motors (Goldstein and Yang, 2000). These depictions revealed significant anterograde bias of HhC particles, with 93.5% of total particles moving toward the motor neuron axon terminal and 86.6% of total particles moving anterogradely in photoreceptors (Fig. 3E and Movies 1 and 2, respectively).

Visibly more Hh was transported in an anterograde direction than retrogradely (Fig. 3). Quantification analysis of photoreceptor neurons corroborates this, with anterograde flux being several-fold higher than retrograde flux (0.54 and 0.07 particles s^−1^, respectively). Flux is defined as the average number of punctae that cross a certain position in the axon at any given time, and can be measured by counting the number of traces in any one direction that run across a line drawn through the kymograph (e.g. see Fig. 3C).

**Fig. 4.**
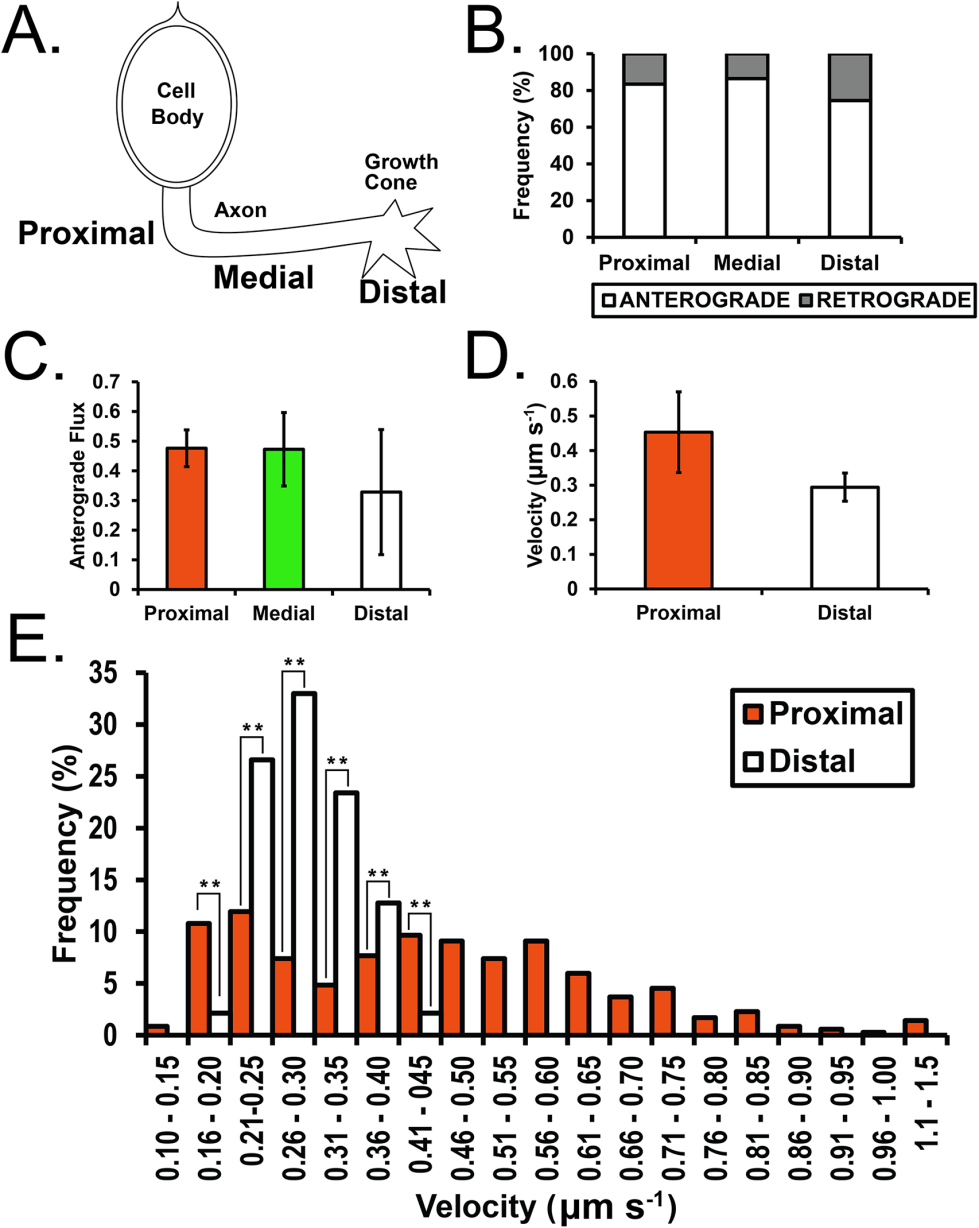
**Positional analysis of HhC transport along *Dmel* photoreceptor axons.** (A) Schematic of an individual photoreceptor cell and proximal, medial and distal axonal position relative to the cell body. (B) Frequency plot of anterograde (white bars) versus retrograde (grey bars) transport in photoreceptor axons. (C) Anterograde flux (particles s^−1^) is lower in the distal axon relative to the proximal and medial axon. (D) Mean velocity (µm s^−1^) measurements for HhC particles in the proximal and distal axon. (E) Velocity frequency distribution for HhC particles in the proximal (orange bars) versus distal (white bars) axon. **P<0.01 by two-tailed *t*-test. Values provided are means from ∼35 min of time lapse data (e.g. proximal, ∼10 min; proximal/medial, ∼10 min; Distal, ∼16 min) and *n* (analyzed traces)=3512 (for proximal, proximal/medial, and distal movies combined in B-D), 351 (for proximal movies alone in E) and 94 (for distal movies alone in E). Error bars are s.e.m.

With this stark disparity in velocity and flux of anterograde versus retrograde particles we wondered whether HhC particles behaved differently depending on whether they were proximal (to the cell body), medial (in the optic stalk) or distal (near the growth cone) (Fig. 4A). While the percentage of anterograde particles was similar in the proximal and medial axon, this number decreases in the distal axon (Fig. 4B and Movies 3 and 4). Indeed, the anterograde flux is most different between the proximal and distal axon (Fig. 4C, Movies 3 and 4). Focusing on just the proximal and distal axon, we found that anterograde HhC particles moved quickly in the proximal axon but then appear to slow down near the growth cone tip (Fig. 4D and Movies 2-4). A plot of the velocities of these particles revealed a ‘two-peak’ distribution in the proximal axon (Fig. 4E, orange bars) with peaks in the ∼0.21-0.25 µm s^−1^ and ∼0.46-0.55 µm s^−1^ ranges, while the distal axon showed just one peak in the ∼0.26-0.30 µm s^−1^ range (Fig. 4E, white bars). These results suggest that there may be two potential modes of anterograde transport for HhC in the proximal axon which appear to resolve into one mode at the distal axon.

Finally, we wondered how Hh might behave in a mutant system. We chose to observe Hh in a recently developed model (in *Drosophila* and mice) of photoreceptor (PR) neurodegeneration, caused by mutations in *fatty acid transport protein* (*fatp*) (Dourlen et al., 2012, 2015)*.* This protein is especially important to Hh processing since it controls the intracellular levels of palmitic acid, a lipid moiety which, in addition to cholesterol, is covalently linked to the mature Hh ligand (Buglino and Resh, 2012; Luiken et al., 1999; Seeßle et al., 2015). Although it is believed that accumulation of either Rhodopsin-1 protein or intracellular ceramide causes the acute adult-onset PR toxicity observed in *fatp* mutants, we wondered if altered Hh transport might also be contributing to the decline in PR health. Importantly, we also chose this model because PR neurodegeneration occurs in the adult eye, and thus the developing PRs are presumed healthy and should be free of any obvious toxic traits that might alter transport (e.g. apoptosis, loss of MT polymerization, damaged mitochondria).

**Fig. 5.**
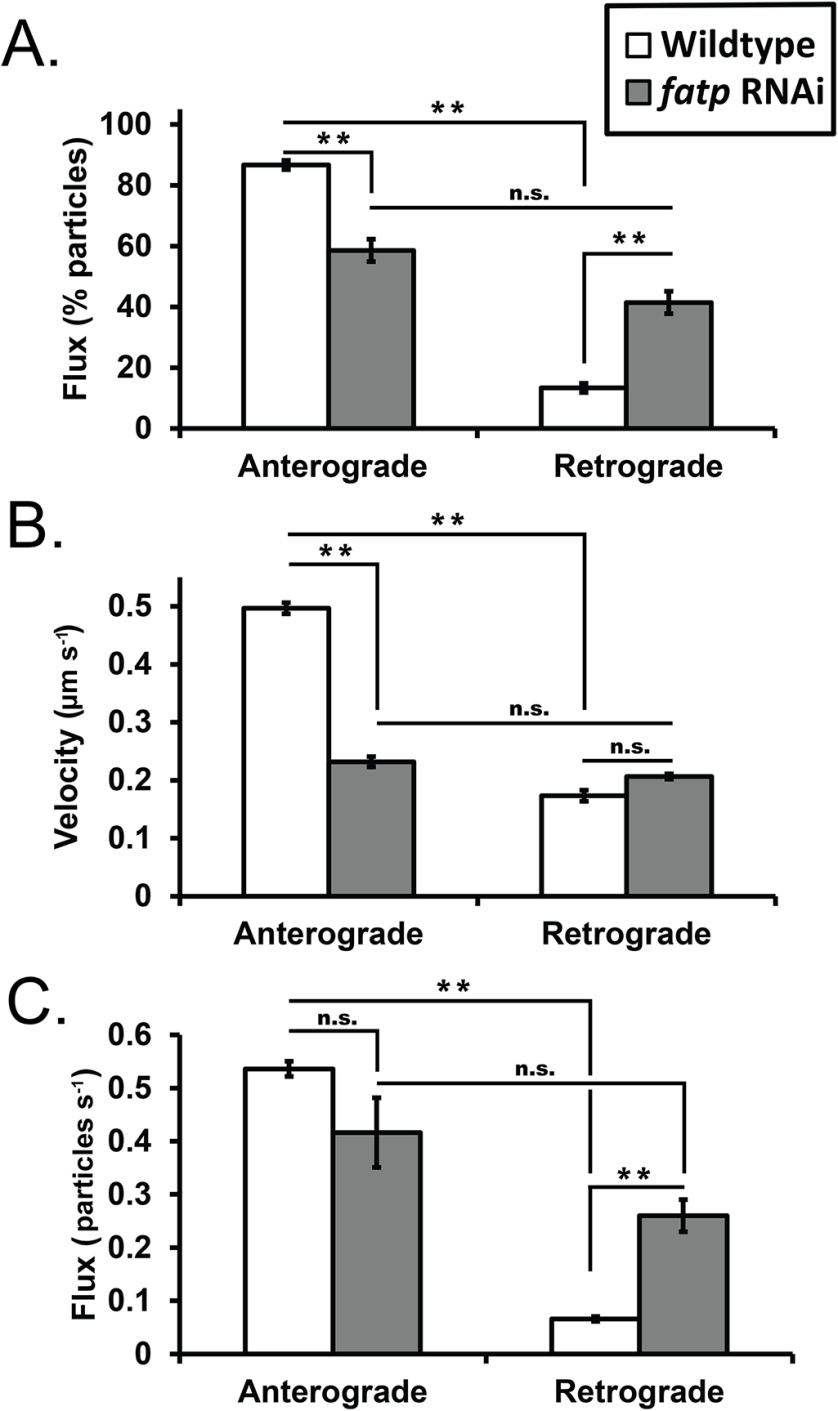
**Characterization of Hh transport in a model of neurodegeneration.** (A) Frequency plot of anterograde versus retrograde Hh transport in photoreceptor axons between wild type (white bars) and *fatp* RNAi (grey bars) (under *GMR-GAL4* control) in third instar larvae. (B) Mean velocity (µm s^−1^) measurements and (C) flux (particles s^−1^) for HhC particles in the anterograde and retrograde direction. Values provided are means from ∼13 min of time-lapse data [*n*(analyzed traces)=405] in the wild-type condition (*GMR-Gal4*>*UAS-ss::GFP::HhC*) and ∼7 min of time lapse data [*n*(analyzed traces)= 210] in the ‘mutant’ condition (*GMR-Gal4*>*UAS-ss::GFP::HhC; UAS-fatp* RNAi). *P<0.05 and **P<0.01 by two-tailed *t*-test. Error bars are s.e.m.; n.s., not significant.

Indeed, Hh flux was significantly different in the PRs of animals driving *fatp* RNAi with 28% of particles shifting to the retrograde direction of transport (ant/ret for: WT, 87%/13%; *fatp* RNAi, 59%/41%) (Fig. 5A). Although the retrograde velocity of these particles remained unchanged (WT, 0.17 µm s^−1^; *fatp* RNAi, 0.21 µm s^−1^), the anterograde velocity was dramatically different in the mutant condition (WT, 0.50 µm s^−1^; *fatp RNAi*, 0.23 µm s^−1^) (Fig. 5B). Possibly the most dramatic difference we observed was the nearly 4× increase in the number of particles moving in the retrograde direction (WT, 0.07 particles s^−1^; *fatp RNAi*, 0.26 particles s^−1^), while anterograde flux was not significantly different (Fig. 5C). Notably, no significant differences in Hh localization were observed with conventional immunohistochemistry (Fig. S1), which demonstrates the unique benefits of dynamic versus static data. Thus, despite PR death being reported exclusively in adult *fatp* mutants (Dourlen et al., 2012), we describe here, for the first time, that altered transport of free fatty acids (FFAs) could also alter Hh transport in the early stages of PR development.

## DISCUSSION

The *Drosophila melanogaster* (*Dmel*) eye is a powerful system to study mechanisms of development, intracellular signaling, behavior, and neurodegenerative disease. Few studies have used dynamic data in the *Dmel* eye to characterize the pathology of neurodegeneration, in particular. By applying live imaging methods in photoreceptors and motor neurons, we were able to characterize transport of the morphogen Hedgehog (Hh), a protein integral to eye-brain development and implicated in stem cell maintenance and neoplastic disease. As an example, we demonstrate the facility of the *Dmel* visual system to derive unprecedented positional and temporal detail of complex phenotypes in real time. We posit that this method could be used to characterize the behavior of pathologically relevant cargoes or organelles in existing models of neurodegeneration or in screening for new phenotypes in disease models that are already characterized (e.g. PINK1, polyglutamine-containing proteins, Lis1) (Liu et al., 2000; Stowers et al., 2002; Wang et al., 2011; Wyan-Ching Mimi Lee et al., 2004).

In fly larval photoreceptor neurons the morphogen Hh is simultaneously released apically to activate ommatidial development and basally, down photoreceptor axons, to the brain to induce the specification of postsynaptic target neurons (Fig. 1). This balance of apical/basal transport to two receptive fields is integral to the precise timing and specification of the *Dmel* eye and brain, though little is known about the mechanisms governing this process. Unlike the other models of Hh in development Hh transport can be comprehensively quantified in various *Dmel* neuron types (Fig. 2). Our dynamic data of neuronal Hh suggests distinct transport kinetics and the possibility of two modes of Hh transport in the proximal axon, which appear to resolve distally (Figs 3 and 4). While little is known about the intracellular movement of Hh in cells that produce the ligand, our bimodal velocity analysis in the proximal axon may be indicative of differential trafficking and secretion of ‘long-range’ and ‘short-range’ Hh complexes, or perhaps of ‘early’ and ‘late’ stages of Hh maturation (Guerrero and Kornberg, 2014).

Unlike previous studies of photoreceptor axon transport (e.g. behavioral readouts and retina/growth cone ratios in the brains of live or fixed animals), we have demonstrated the ability to directly visualize transport in real time in intact and live animals, and the option to convert 3D time-lapse data into a 2D kymograph (Liu et al., 2000; Stowers et al., 2002; Wyan-Ching Mimi Lee et al., 2004). Kymographs, which are routinely applied to mitochondrial axon transport, can be a great source of information as they reveal subtle nuances in the transport behavior of cargo (Baqri et al., 2009). Precise analytical details such as direction, volume and velocity that are difficult to derive from movies can be conveniently quantified from kymographs. The direction of traces reflects directionality of transport. For example, Hh-positive puncta that move in the anterograde direction trace a forward trajectory and vice-versa for retrograde transport. Therefore, even the most abrupt transient reversals in transport are easily detected on kymographs. Such fine analysis of transport is inconceivable using techniques such as immunohistochemistry and western blotting that have been traditionally applied to Hedgehog transport studies.

Our method also offers a unique opportunity to characterize how disruption of cell biological components (endoplasmic reticulum, Golgi, endocytosis) or mutation of specific residues in Hh (e.g. mutations in holoprosencephaly) affect transport. Since developing *Dmel* photoreceptors resemble neurons (Liu et al., 2000; Wyan-Ching Mimi Lee et al., 2004), it is also possible to use this technique to complement a screen for regulators of Hh axonal transport to see which larvae have enhanced transport of Hh to the brain or the eye (Stowers et al., 2002). More broadly, our method can be applied to characterize the movement and directionality of other biologically important cargoes (e.g. presynaptic markers, labeled Golgi or mitochondria) implicated in development or in various disease models.

We also characterized Hh transport in a developed model of photoreceptor (PR) neurodegeneration. Gene knockdown of *fatp* was chosen because of Fatp's role in the transport of palmitic acid, a fatty acid that is covalently attached (in addition to cholesterol) to create mature Hh ligand. In our characterization (Fig. 5) we observed dramatically reduced anterograde transport of Hh, accompanied by a ∼2.1× decrease in anterograde velocity. Retrograde transport also increased ∼3.1× relative to wild type with a concomitant increase in retrograde flux (∼3.9×). These results suggest that less ‘mature’, lipidated Hh is being sent down axons and that a large proportion of Hh that reaches growth cones is being sent back, since *fatp* RNAi larvae exhibited slower-moving, lower flux, anterograde Hh. Notably, we do not believe that defective secretion is causing the aberrant transport of Hh since another post-translationally lipidated, membrane-targeted protein, Rhodopsin-1, appears to properly localize and oligomerize in *fatp* mutant *Drosophila* (Dourlen et al., 2012; Kock et al., 2009; O'Brien and Zatz, 1984). Thus, we have identified a novel mode of PR toxicity which is perceptible long before PR death occurs. This finding would not have been apparent if only static data had been used.

Finally, it should be noted that a number of *Dmel* neurodegenerative disease models exist in tissues other than the eye (e.g. motor neurons, mushroom bodies), but the dynamic characterization of axon transport in these models involves *ex vivo* culturing of dissected brains (Medioni et al., 2015; Millecamps and Julien, 2013; Rabinovich et al., 2015; Sinadinos et al., 2009; Vos et al., 2008; Williamson and Hiesinger, 2010). While these techniques are excellent and will likely yield many new insights, they are labor-intensive and thus, less amenable to use in genetic screens. Our method, by contrast, requires minimal preparation and allows the complement of adult eye phenotypes and live imaging of intact larvae.

## CONCLUSIONS

We demonstrate the ability to comprehensively quantify the movement and directionality of a developmentally important cargo in neurons during eye-brain development in a living organism in healthy and mutant conditions. This enables us to ask questions regarding the directionality, velocity, and flux of particles and investigate their behavior relative to axonal position. We posit that this method could be adapted to neurodegenerative disease models (e.g. in the *Dmel* eye) and allow future research to characterize the role of important cargoes (e.g. secreted ligands, organelles) and candidate drugs in the fields of cell polarity, axonal transport, and eye-brain development in any practical, transparent, and genetically tractable model system.

## MATERIALS AND METHODS

### Strains

The *UAS-ssGFP-HhC* was described by Chu et al. (2006) and the *UAS-hh^NHA^* by Burke et al. (1999). Additional stocks were obtained from the Bloomington *Drosophila* Stock Center (Bloomington, IN, USA): *y,w; GMR-GAL4/CyO, yw, elav-GAL4 (X),* and *y, w; Bl^1^/CyO, y^+^; CCAP-GAL4;* or from the Vienna *Drosophila* Resource Center (Vienna, AT): *y, w; UAS-P{attP,y+,w3'}VIE-260B* (transformant ID: 100124, construct ID: 104809) which encodes a hairpin against *dFatp* (CG7400).

### Immunohistochemistry

Antibody staining, specimen mounting and microscopy was performed as previously described (Huang and Kunes, 1996). Antibodies were used at the following dilutions: mouse anti-Chaoptin (24B10, DSHB, Iowa City, IA, USA; 1:20), rabbit anti-HA (sc-805, Santa Cruz, Dallas, TX, USA; 1:400), Cy3-goat anti-rabbit (111-165-045, Jackson, Bar Harbor, ME, USA; 1:100) and Cy5-goat anti-mouse (115-175-146, Jackson; 1:100). Frozen glycerol aliquots of these antibodies were from the same batch as those used in Chu et al. (2006) and Dearborn et al. (2002).

### Time-lapse imaging

Crawling third-instar larvae expressing fluorescently labeled HhC (ss::GFP::HhC) (Chu et al., 2006) were anaesthetized in halocarbon oil with 15% chloroform, sufficient to inhibit muscular contraction. Larvae were mounted between a slide and coverslip, and stretched under pressure to bring nerves closer to the cuticle (Fig. 2A,B). Images were acquired with a 63× PlanApo oil objective, NA 1.4, on a Zeiss 7 Live upright scanning confocal with a CCD detector. Images were captured at 0.5 Hz, for a total elapsed time of ∼3-8 min at ∼25°C. All methodology and statistics (including choice of sample size, exclusion criteria, double blind test, randomization, and choice of statistical test) were performed as previously described and according to standard procedures for this type of *Drosophila* live-imaging data (Baqri et al., 2009; Miller and Sheetz, 2006). A detailed description of this preparation is included below.

### Analysis

To generate kymographs, time-lapse frames were aligned horizontally with cell body on the left and axon terminal on the right, re-sliced and *z*-projected. Kymographs in Fig. 3B,C were color inverted in Adobe Photoshop (Adobe, San Jose, CA, USA) to facilitate visibility of transport events. For quantification of anterograde and retrograde transport, total numbers of transport events in each direction were counted at two different positions in each axon and averaged. For velocity of transport, lines were hand-traced over the path of moving punctae. The slope was calculated for each case.

For Hh distribution analysis in Fig. S1, specimens were viewed on a Zeiss LSM700 Inverted confocal microscope 40× PlanNeofluar oil objective, NA 1.3, with constant acquisition settings when comparing specimens within a given experiment. Quantification of growth cone, optic stalk and eye disc fluorescence was performed with Image J as described previously (Chu et al., 2006). Growth cone/optic stalk, eye disc/optic stalk, and growth cone/eye disc average fluorescence ratios were computed as in (Chu et al., 2006) as well. A detailed description of this preparation is also included at the end of the methods section.

### Step-by-step method for data acquisition and analysis

#### Reagents

1. 25×95 mm vial with standard fly food (cornmeal and agar) to maintain parent cross/genotype.
2. Fine-tipped paintbrushes, Petri dish with distilled water, and tissue to collect, rinse and dry the larva.
3. 5 ml of 15% chloroform in halocarbon oil 700 for anesthesia.
4. Glass slide, coverslips and double-sided sticky tape for mounting the larva.

#### Selecting sample

1. Collect flies of desired genotype/cross in vial with standard fly food.
2. Transfer flies to fresh vial after 6 h.
3. Maintain eggs collected over the 6 h period in first vial.
4. Grow at 25°C with 60% humidity and 12 h light:dark cycles.
5. 90 h after egg laying, pick larvae that are freely crawling on the walls of the vial with soft brush.

#### Preparing the mount

1. Cut 4 strips of double-sided sticky tape, 0.5 cm wide.
2. Place glass slide on flat surface and paste two layers of tape, one on top of another. Paste the remaining two strips in the same fashion approximately 2 cm away. Set the slide aside.
3. Place glass coverslip under light microscope and add a drop of 15% chloroform in the center.

#### Preparing the sample

1. Transfer larva directly to Petri dish with distilled water at room temperature.
2. Gently wash larva in water to remove food and debris that may be stuck to body.
3. Blot it gently on tissue until dry.
4. Transfer larva to the drop of chloroform on the coverslip under the light microscope.
5. Align larva laterally (Fig. 2A,B), such that larva is lying on side, ∼45° from the spiracles.
6. Roll the larva gently with brush a few times to make sure the larva does not curl up.
7. Gently place slide on the coverslip, such that the chloroform-engulfed larva lies in between the adjacent strips of double sided sticky tape (Fig. 2B).
8. Press sides down once, and then quickly flip around the slide to ensure that coverslip now faces up.
9. Use the back of a brush to press down on sides of coverslip. Make sure that tape is securely stuck and there are no air vacuoles in between tape and coverslip. This allows the larva to stretch to an extent where the photoreceptor axons are pushed out and come closer to the cuticle.

#### Image acquisition

1. While this protocol utilizes the upright Zeiss 7 Live microscope, similar time-lapse movies can be acquired on any fast-scanning microscope capable of scan speeds faster than 5 frames per second.
2. 63× oil lens, NA 1.4 works well to capture the desired field of view as well offer optimum magnification.
3. Images in the time-lapse series are acquired at 2 s intervals, for a total of 300 s.

#### Image analysis

1. Open file in ImageJ.
2. Crop image to appropriate size.
3. Run the plugin ‘Stackreg’ with ‘Rigid Body’ selection in the dropdown menu. This step will align the image to compensate for shift in the *x-y* plane during imaging.
4. Rotate image appropriate amount to align the photoreceptor nerve such that cell body is on the left and axon terminal on the right. Use the ‘Rotate’ feature of ‘TransformJ’ and apply cubic-b-spline interpolation.
5. Reslice image, with output spacing set to 1 µm. Start at the top and avoid interpolation by using 1 pixel spacing.
6. Make a *z*-projection image of the resliced file, using the maximum intensity projection, to generate kymograph of net motion.
7. Save as tiff file. Open in Adobe Photoshop, covert to 8-bits/pixel, adjust levels to appropriate extent, and invert color to better visualize individual transport events.
